# Omalizumab treatment for chronic spontaneous urticaria coinciding with marked improvement of vitiligo: a case report

**DOI:** 10.3389/fmed.2026.1752774

**Published:** 2026-08-19

**Authors:** Songfa Lin, Pengjun Zhou, Shaoming Huang, Kunjie Li, Liangliang Zhang

**Affiliations:** 1 Department of Dermatology, the Second Affiliated Hospital of Fujian Medical University, Quanzhou, Fujian, China; 2 Department of Dermatology, the Hospital of Nan’an City in Fujian Province, Quanzhou, Fujian, China

**Keywords:** chronic spontaneous urticaria, immunotherapy, omalizumab, repigmentation, vitiligo

## Abstract

We report the case of a 49-year-old female patient with a history of hyperthyroidism and long-standing vitiligo for over two decades, who presented with a 6-year history of chronic spontaneous urticaria (CSU) refractory to conventional antihistamine therapy. She was subsequently initiated on subcutaneous omalizumab. This treatment not only led to significant control of her urticaria but also resulted in the unexpected observation of repigmentation of vitiligo lesions on the face and neck. As of the last follow-up in July 2025, the patient remained on omalizumab therapy with sustained improvement in both conditions. This case highlights a potential novel therapeutic effect of omalizumab on vitiligo, warranting further clinical and mechanistic investigation.

## Introduction

Chronic spontaneous urticaria (CSU) is a complex, mast cell-driven disorder characterized by the recurrent appearance of pruritic wheals and/or angioedema persisting for more than 6 weeks (1). Omalizumab, a humanized monoclonal antibody targeting immunoglobulin E (IgE), is an established therapy for antihistamine-refractory CSU. It reduces free IgE levels and downregulates FcεRI receptors on mast cells and basophils, thereby inhibiting their activation (2). Vitiligo, in contrast, is an autoimmune depigmenting disorder in which melanocytes are targeted and destroyed by autoreactive T cells, leading to significant psychosocial comorbidity (3). Interestingly, there are anecdotal reports of vitiligo repigmentation following treatment with mast cell-stabilizing agents such as ketotifen (4, 5). However, to the best of our knowledge, there are no prior published reports documenting the effect of omalizumab on vitiligo. In this study, we describe a case of concurrent improvement in CSU and vitiligo following omalizumab therapy, exploring the potential immunological interplay.

## Case report

**Figure 1:**
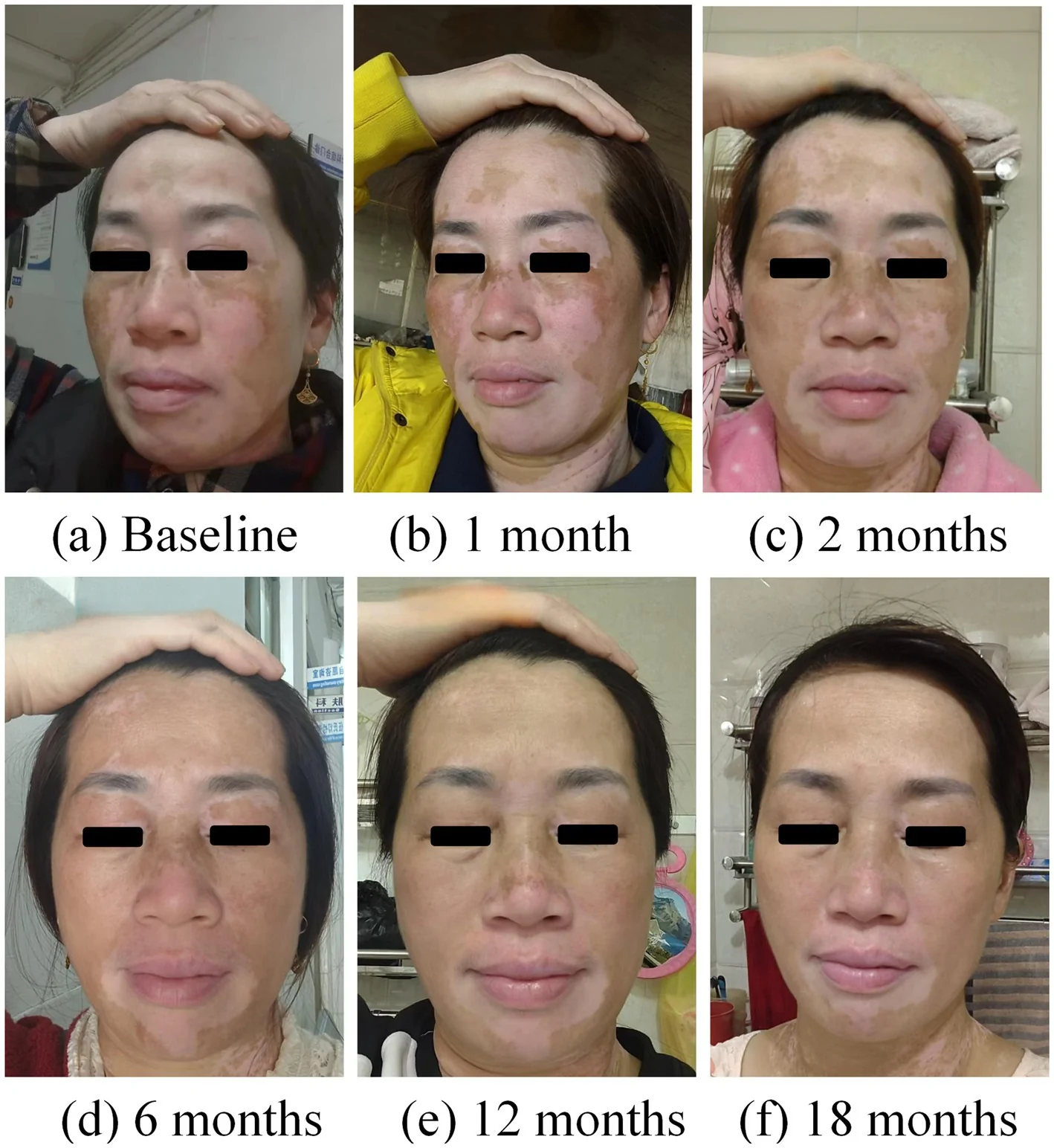
Serial changes in vitiligo lesions on the face and neck of the patient following omalizumab treatment. The respective time points and corresponding Vitiligo Extent Score for a Target Area (VESTA) are as follows: **(a)** Before treatment, baseline; **(b)** after 1 month of treatment, VESTA score 14.5%; **(c)** after 2 months of treatment, VESTA score 40.2%; **(d)** after 6 months of treatment, VESTA score 60.3%; **(e)** after 12 months of treatment, VESTA score 70.3%; and **(f)** after 18 months of treatment, VESTA score 80.2%.

We describe the case of a 49-year-old female patient who presented to our clinic in December 2023 with a 6-year history of recurrent, generalized wheals accompanied by intense pruritus. The lesions were typical of urticaria, exhibiting rapid evolution and complete resolution without residual marks. She had been diagnosed with CSU and showed a suboptimal response to sequential trials of various H1-antihistamines, including loratadine and levocetirizine. Her past medical history was significant for hyperthyroidism, well controlled on a maintenance dose of methimazole (5 mg daily), and vitiligo of over 20 years’ duration. The patient had previously received topical corticosteroids and calcineurin inhibitors with limited efficacy, and treatment records were incomplete; she had discontinued all treatments for over 3 years. Cutaneous examination at presentation revealed extensive, non-scaly, depigmented macules and patches distributed over the face, neck, trunk, and limbs, covering approximately 50% of the body surface area (Figure 1a). In light of her refractory CSU, treatment with subcutaneous omalizumab 300 mg monthly was commenced in December 2023. Following the first two doses, a marked reduction in the frequency and severity of urticaria episodes was noted, along with significant alleviation of pruritus. Remarkably, during the initial 1–2 months of therapy, the patient reported the onset of punctate and patchy repigmentation within the vitiliginous patches on her face and neck. This was objectively quantified using the Vitiligo Extent Score for a Target Area (VESTA), which showed improvements from baseline to 14.5 and 40.2% in representative lesions (Figures 1b,c). The vitiligo on her trunk and limbs remained largely unchanged. With continued omalizumab treatment, her urticaria remained well-controlled. Sequential follow-up demonstrated progressive repigmentation in the facial and neck lesions at 6, 12, and 18 months, with VESTA scores further improving to 60.3, 70.3, and 80.2%, respectively (Figures 1d–f). At the most recent follow-up in July 2025, her urticaria was in complete remission, and the repigmentation of facial lesions was sustained, while lesions elsewhere remained stable. The therapy was well-tolerated without any adverse events reported.

## Discussion

This case illustrates a compelling dual response to omalizumab: effective control of refractory CSU and a concurrent, significant improvement in vitiligo. The primary mechanism of omalizumab in CSU involves the sequestration of free IgE and the subsequent downregulation of the high-affinity IgE receptor (FcεRI) on effector cells, blunting mast cell and basophil degranulation (2). Vitiligo pathogenesis is primarily attributed to T-cell-mediated autoimmunity against melanocytes (3).

The observed vitiligo repigmentation suggests that omalizumab may exert immunomodulatory effects beyond its known role in urticaria. We hypothesize several non-mutually exclusive mechanisms. First, mast cells, whose activity is modulated by omalizumab, are resident in the skin and may contribute to the local inflammatory milieu in vitiligo by releasing cytokines and chemokines that recruit or activate autoreactive T cells (6, 7). Second, there is emerging evidence that IgE itself may play a direct role in autoimmunity; by intercepting IgE, omalizumab might disrupt aberrant IgE-mediated signaling that could potentially influence T-cell activation or autoantibody production (8). Third, omalizumab treatment in CSU is associated with reduced IgE levels, and increasing evidence has suggested that CSU is associated with the activation of coagulation-related and immunothrombotic pathways. These findings imply that omalizumab may have broader anti-inflammatory and immunomodulatory effects in addition to its classical anti-allergic mechanism. Fourth, omalizumab treatment in CSU is associated with reduced IgE levels, and increasing evidence has suggested that CSU is associated with the activation of the coagulation cascade. These findings imply that omalizumab may have broader anti-inflammatory effects in addition to classical allergic mechanisms (9). Further support for a broader role of omalizumab in autoimmune dermatologic disease comes from reports of its efficacy in bullous pemphigoid, an IgE-associated autoimmune blistering disorder (10, 11). Nevertheless, these mechanisms remain speculative, and the significance of biomarkers in predicting or explaining omalizumab responsiveness remains incompletely defined. Therefore, causality should not be overinterpreted on the basis of a single case report.

The localized response on the face and neck likely reflects differences in melanocyte reservoirs and local immune microenvironment, with greater repigmentation in areas of richer follicular reserves. The temporal association and the objective VESTA score documentation strengthen the likelihood of a causal relationship. However, spontaneous repigmentation is well documented in vitiligo and remains an important confounding factor. Future reports would be strengthened by longer follow-up, serial biomarker assessment such as total IgE and D-dimer, and, where feasible, histopathological or immunologic correlates. Prospective, controlled studies are required to validate this observation and elucidate the underlying mechanisms.

## Conclusion

This report provides novel evidence that omalizumab may confer a beneficial effect on vitiligo in addition to its intended action on CSU. This serendipitous finding suggests a potential repurposing of this biologic agent for a challenging autoimmune dermatosis. Clinicians should be observant of such effects in their practice. Further investigative efforts are strongly encouraged to confirm the efficacy of omalizumab in vitiligo and to delineate its mechanism of action.

## Data Availability

The original contributions presented in the study are included in the article/supplementary material, further inquiries can be directed to the corresponding author.
